# Congenital Lobar Emphysema in Children: Case Series

**DOI:** 10.7759/cureus.49416

**Published:** 2023-11-26

**Authors:** Nada Benbouziane, Loubna Larda, Christian Pongo, Fatima zahra Alaoui-Inboui, Bouchra Slaoui

**Affiliations:** 1 Pediatrics, Hôpital Mère-Enfant Abderrahim Harouchi, Casablanca, MAR; 2 Pediatrics and Child Health, Hôpital Mère-Enfant Abderrahim Harouchi, Casablanca, MAR

**Keywords:** pulmonary lobectomy, congenital lung malformations, recurrent wheezing, neonatal respiratory distress, congenital lobar emphysema

## Abstract

Recurrent wheezing is very common in infants. When these symptoms appear early without a free interval, a pulmonary malformation should be investigated. Congenital lobar emphysema is a rare abnormality of the lower respiratory tract. Here, we report a case series of six cases of congenital lobar emphysema between 2015 and 2023. Clinical and radiological data were collected according to an operating sheet previously established in our pediatric pneumo-allergology unit. They all had recurrent wheezing and dyspnea. Chest radiography and chest CT were consistent with the diagnosis of congenital lobar emphysema. All patients had lobectomy without complications.

## Introduction

Recurrent wheezing is common in infants and requires a good etiological approach in order to make the diagnosis. Their early onset and the absence of a free interval between wheezing episodes are key elements that can point toward malformations, including congenital lobar emphysema (CLE). It is one of the most common bronchopulmonary malformations characterized by distention of a pulmonary lobe without destruction of the parenchyma. It manifests as respiratory distress due to excessive expansion of one or more pulmonary lobes of the lung with compression of the underlying lobe. This results in air being trapped during the expiratory phase, causing recurrent respiratory distress, most often detected in the neonatal period or later in childhood [1]. The aim of this case series is to describe the clinical symptoms of revelation and the radiological signs of CLE.

## Materials and methods

This is a case series of six cases of CLE, collected over a period of eight years (from January 2015 to September 2023) using the data from their medical files. We included all infants hospitalized for persistent wheezing that started in the neonatal period with scannographic confirmation of CLE (six cases). We collected anamnestic data: age, sex, age of onset of symptoms, type of symptoms: cough, wheezing, respiratory distress, respiratory infections, and treatments received before hospitalization. The clinical examination looks at the presence of cyanosis, respiratory rate, signs of respiratory distress, wheezing or crackles, fever, and weight. The oxygen saturation on room air was measured for all patients upon admission and during hospitalization. A chest x-ray and a chest CT scan were performed for all patients. As soon as the diagnosis was confirmed radiologically, the patients were all entrusted to pediatric surgeons in order to proceed with the surgical treatment.

## Results

**Figure 1:**
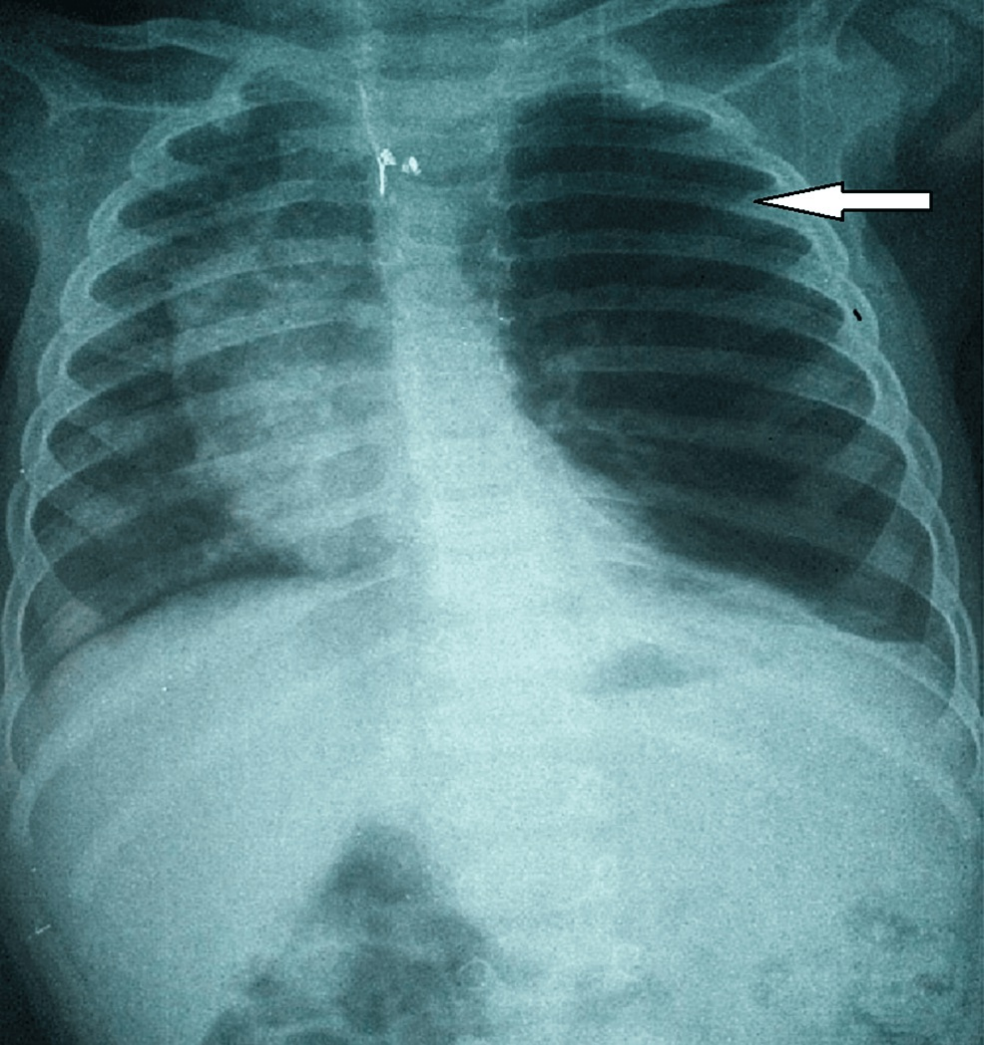
Chest x-ray showing hyperlucency of the left pulmonary field and repression of the mediastinum on the right side with lower lobe atelectasis.

**Figure 2:**
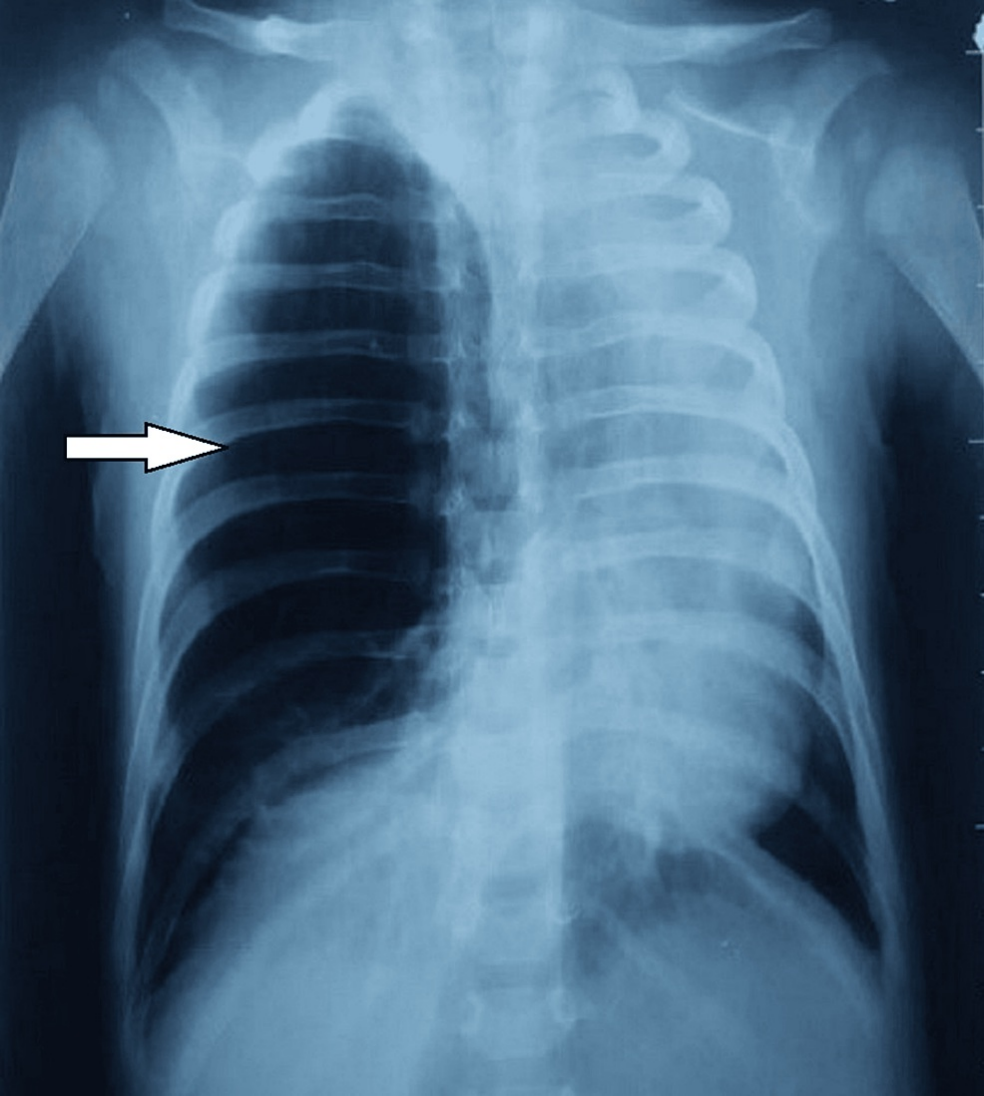
Chest x-ray of the first patient showing hyperlucent right hemithorax.

**Figure 3:**
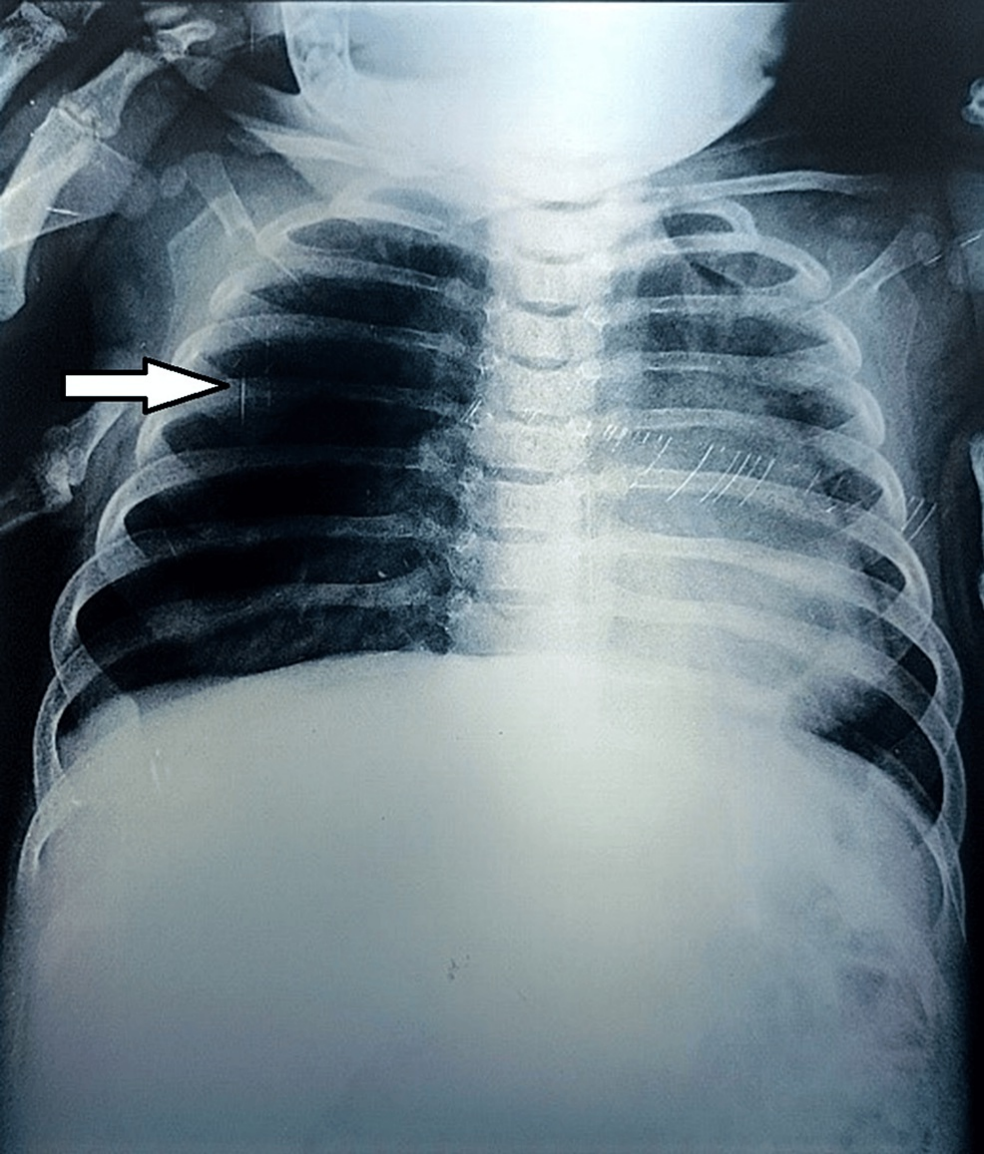
Chest x-ray of the second patient showing hyperlucent right hemithorax. Hyperlucent right hemithorax

There are 50% of the patients were male. Aged from four months to 17 months with an average age of nine months. The age of onset of symptoms was neonatal. Two patients were previously treated for childhood asthma with inhaled corticosteroids. The clinical manifestations during hospitalization are represented by recurrent wheezing and dyspnea (six cases), intermittent cyanosis (two cases), pulmonary infection (two cases), and a dry hacking cough (one case) (Table 1). Physical examination revealed respiratory distress, tachypnea, and wheezing in all patients. Chest radiography revealed hyperlucency of the left upper lobe in three cases (Figure 1) and the right upper lobe with atelectasis of the lower lobe in three cases (Figures 2, 3), and one case an association with a mediastinal hernia with deviation of the mediastinum toward the contralateral side (Figure 2).

**Table 1:** Clinical manifestations of the six cases of congenital lobar emphysema

Results	First case	Second case	Third case	Fourth case	Fifth case	Sixth case
Sex	Male	Male	Female	Male	Female	Female
age	8 months	11 months	8 months	4 months	17 months	6 months
Medical background	Hacking cough,Dyspnea from birth, an hospitalization at 6months old and treated as asthma.	Dyspnea from birth, an hospitalization at 3days old and treated as asthma at 6 months old	Dyspnea from birth	Cyanosis, Dyspnea from birth and fatigue during feeding.	Dyspnea from birth	Cyanosis, Dyspnea from birth.
clinical exam	Dyspnea with Respiratory rate (RR) 51 breaths/minute , bilateral wheezing , an oxygen saturation of 99% on room air, fever at 38.3 and weight 7 kg 500 g	Dyspnea, RR 60 breaths/minute, bilateral wheezing , oxygen saturation of 94% on room air, weight 9 kg and without fever	Dyspnea, RR 76 breaths/minute, bilateral wheezing , oxygen saturation of 90% on room air, weight 5 kg 600 g and without fever	Dyspnea, RR 65 breaths/minute , bilateral wheezing, oxygen saturation of 96% on room air , weight 6 kg 600 g and without fever	Dyspnea, RR 52 breaths/minute, bilateral wheezing, oxygen saturation of 94% on room air,without fever and weight 10 kg	Dyspnea, RR 65 breaths/minute, bilateral wheezing, oxygen saturation of 94% on room air, fever 38.6 and weight 9 kg

**Figure 4:**
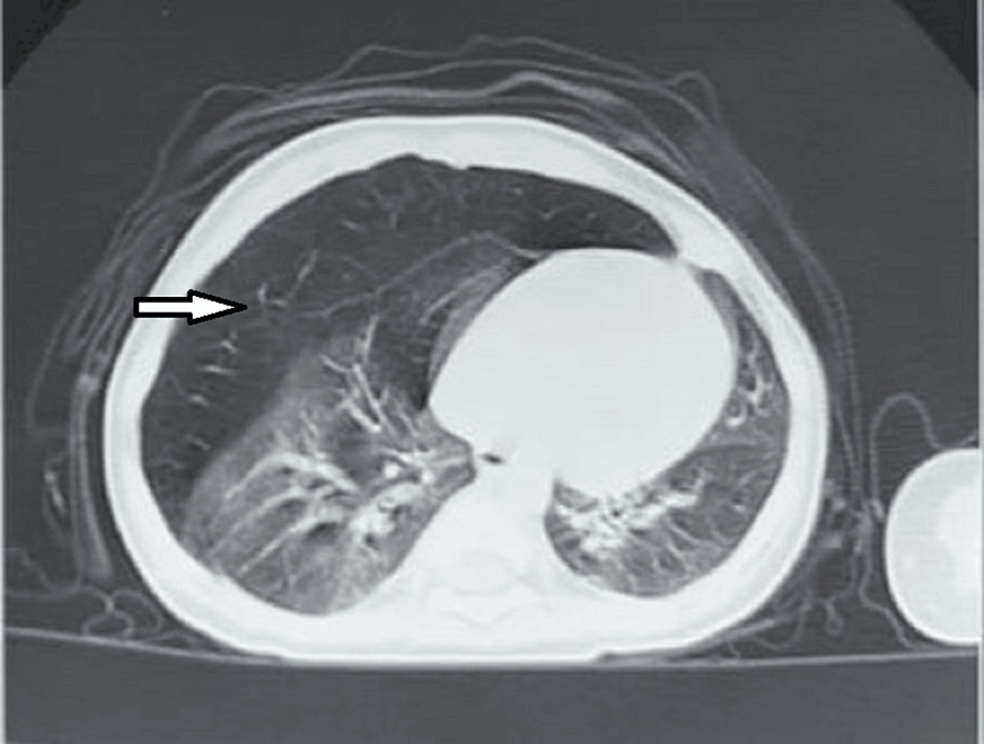
Chest CT showing right lobar emphysema

**Figure 5:**
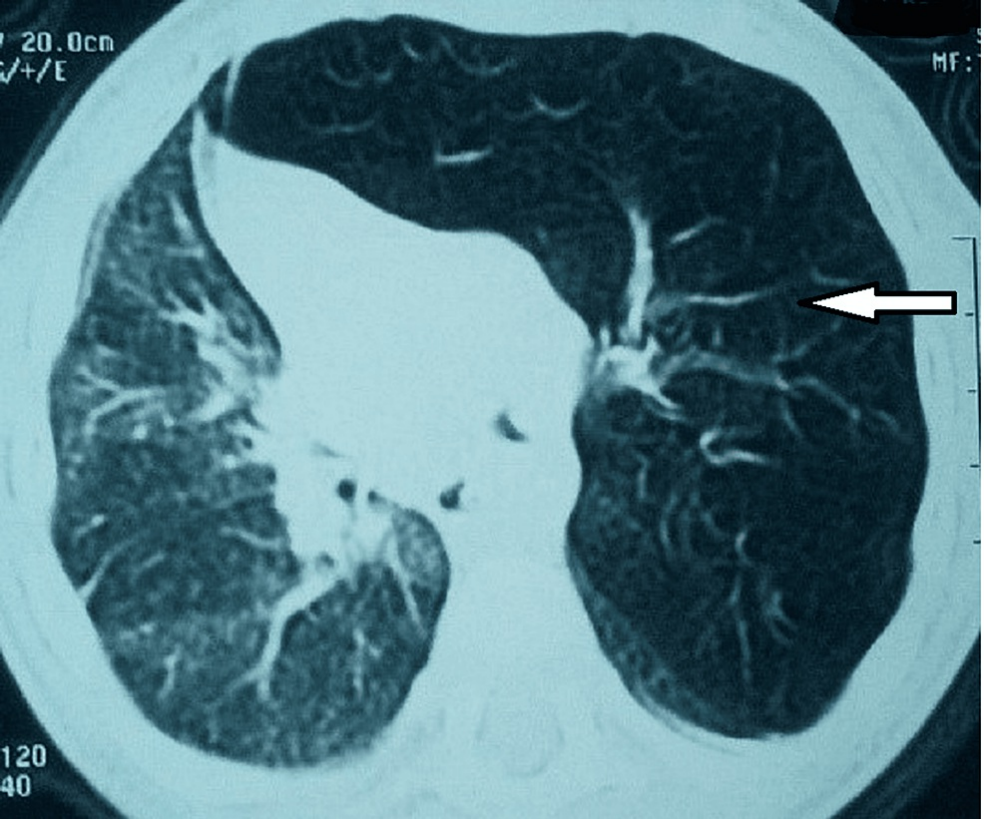
Chest CT showing giant left lobar emphysema.

A chest CT scan confirmed the diagnosis, showing hypodensity on the affected side with rarefaction of the vascular elements on the right side (Figure 4) and left side (Figure 5). All six patients benefited from lobectomy of the emphysematous lobe with good clinical and radiological improvement.

## Discussion

Congenital bronchopulmonary malformations are rare, their incidence is estimated at 3.4/10,000 births. CLE represents approximately 14% of all congenital bronchopulmonary malformations in children [2-5], and its prevalence is estimated at 1/20,000 to 30,000 births with a male predominance [6]. CLE is rarely diagnosed antenatally. It is characterized by the progressive distention of one lobe, sometimes of two lobes. It can be associated with various malformations (Table 2) including cardiac malformations in 14%-20% of cases [7], or more rarely with pulmonary sequestration [8]. In its typical form, CLE most often affects the left upper lobe [9].

**Table 2:** Associated malformations with the congenital lobar emphysema

Malformations	Type
Cardiac malformations 14%-20%	Ductus arteriosus, Atrial septal defect, Ventricular septal defect, Tetralogy of fallot, Pulmonary stenosis, Pulmonary valve atresia, Aortic coarctation
Renal anomalies	Aplasic kidney, Horseshoe kidney
Musculoskeletal anomalies	Hiatal hernia, Diaphracmatic hernia
Gastrointestinal tract	Omphalocele, Pyloric stenosis

The etiology of CLE remains undetermined in half of the cases. In 25% of cases, congenital emphysema results either from the absence of bronchial cartilage or from hypoplasia or dysplasia of this cartilage. This cartilage deficiency leads to a weakening of the bronchial wall and then bronchial collapse leading to air trapping during expiration [10].

CLE can be symptomatic in the neonatal period but is most often discovered from one to six months. It manifests itself by recurrent wheezing, cyanosis, wheezing, whining, and in other more severe cases tachypnea and acute respiratory failure [11]. In the study of Cataneo et al., respiratory symptoms were present in 60% of cases from birth [9]. Dyspnea was the most common symptom, which is the same as our study. CLE is rarely asymptomatic and discovered incidentally in older children or even adults [12,13].

Dyspnea often develops gradually, evolving in afebrile patients, suggesting a malformative origin. If diagnosis is delayed, it can progress to respiratory distress due to compression of adjacent structures, which can be life-threatening [14,15]. Abdel Bary et al. reported wheezing dyspnea in 59% of cases in a study including 37 cases of CLE [16]. Recurrent pulmonary infection was reported in 38% and 48% of cases, respectively, in the Cataneo and Abdel Bary series, thus potentially delaying the diagnosis [9,16]. Two of our patients were subject to recurrent pulmonary infections.

A chest x-ray is the first additional examination to be performed. The chest x-ray shows a distended, hyperlucent affected lobe containing a fine vascular network. This distension pushes back the mediastinum, therefore compressing the ipsilateral lobe, as is the case for all patients. Chest CT remains the most specific exam to establish the diagnosis and the topography. The left upper lobe is most often affected (43%), followed by involvement of the right middle lobe (32%) and the right upper lobe (21%) [17]. The lower lobes are rarely affected, with less than 1%. Furthermore, a few cases of bilateral ELG have been described in the literature but this remains rare [17].

In this small case series, all patients underwent chest CT scanning revealing congenital right and left lobar emphysema in three cases each. The MRI with three-dimensional and vascular sequences is the alternative choice without the injection of contrast product. It helps to diagnose the malformations with a vascular component [17]. In certain atypical cases, bronchoscopy is indicated to eliminate the presence of an intrabronchial foreign body, or a mucous plug or to look for a bronchial anomaly that could be responsible for emphysema. Lung scintigraphy is an important examination that allows visualization of ventilation and perfusion disorders in the diseased area. It can also provide information to differentiate CLE from compensatory emphysema [13]. The definitive diagnosis, however, remains the pathological examination after surgical excision which remains the usual treatment for CLE. Surgical excision of the emphysematous lobe is carried out more or less urgently depending on the severity of the respiratory distress. This lobectomy allows the reexpansion of the compressed lobe [3,9]. According to Rhotenberg, the optimal age for performing an intervention would be between one and six months [18]. In this series, all infants underwent lobectomy with pathological confirmation. Postoperative complications may include secondary pulmonary arterial hypertension. All patients in our study improved after surgical treatment without complications.

## Conclusions

Although CLE remains a rare entity in pediatrics, it is still a frequent reason for consultation. Due to its rarity, it may be misdiagnosed with other conditions. The diagnosis of CLE is made on the basis of clinical and radiological evidence. Treatment is essentially surgical. It should be noted that the purpose of this case series is to guide the practitioner to make the diagnosis early and ideally in the neonatal period in order to improve the functional prognosis.
